# Characterization of genetic subclonal evolution in pancreatic cancer mouse models

**DOI:** 10.1038/s41467-019-13100-w

**Published:** 2019-11-28

**Authors:** Noushin Niknafs, Yi Zhong, John Alec Moral, Lance Zhang, Melody Xiaoshan Shao, April Lo, Alvin Makohon-Moore, Christine A. Iacobuzio-Donahue, Rachel Karchin

**Affiliations:** 1 Department of Biomedical Engineering, Institute for Computational Medicine, Johns Hopkins University, Baltimore, MD 21218 USA; 2 Sloan Kettering Institute, Memorial Sloan Kettering Cancer Center, New York, NY 10065 USA; 3 Human Oncology and Pathogenesis Program, Memorial Sloan Kettering Cancer Center, New York, NY 10065 USA; 4 Department of Surgery, Memorial Sloan Kettering Cancer Center, New York, NY 10065 USA; 5 Department of Pathology, Memorial Sloan Kettering Cancer Center, New York, NY 10065 USA; 6 David M. Rubenstein Center for Pancreatic Cancer Research, Memorial Sloan Kettering Cancer Center, New York, NY 10065 USA; 7 Department of Oncology, Cancer Biology Program, Johns Hopkins Medical Institutions, Baltimore, MD 21287 USA

**Keywords:** Cancer, Cancer genomics, Cancer models, Gastrointestinal cancer, Tumour heterogeneity

## Abstract

The *KPC* mouse model, driven by the *Kras* and *Trp53* transgenes, is well regarded for faithful recapitulation of human pancreatic cancer biology. However, the extent that this model recapitulates the subclonal evolution of this tumor type is unknown. Here we report evidence of continuing subclonal evolution after tumor initiation that largely reflect copy number alterations that target cellular processes of established significance in human pancreatic cancer. The evolutionary trajectories of the mouse tumors show both linear and branching patterns as well as clonal mixing. We propose the *KPC* model and derivatives have unexplored utility as a functional system to model the mechanisms and modifiers of tumor evolution.

## Abstract (web summary)

In pancreatic cancer the *Kras* and *Trp53* transgene driven *KPC* mouse model is used to experimentally study disease processes. Here, the authors analyse tumour evolution within the *KPC* model, finding both linear and branched evolution and highlighting the utility of this model in mechanistic research of tumour evolution.

## Introduction

Since the introduction of the first genetically engineered mouse model (GEMM) of pancreatic adenocarcinoma (PDA) in 2003^1^, GEMMs have proved to be powerful tools in investigating PDA etiology and development^2–4^. Most GEMMs are established by combining pancreas-specific endogenous expression of a mutant *Kras* oncogene, which is mutated in 95% of human PDA cases, in combination with pancreas-specific endogenous inactivation of one or two tumor suppressors genes (*Cdkn2a*, *Trp53*, *Smad4*, *Tgfbr1*, and Tgfbr2) frequently altered in human PDA. These models have successfully yielded insights into the cell of origin^5,6^, role of the tumor microenvironment^7,8^ and inflammation^9,10^ in PDA, contribution of pancreatitis^9,11,12^, and diagnostic/therapeutic possibilities^1,8,13,14^.

The canonical *KPC* (*LSL-Kras*^*G12D/+*^*; LSL-Trp53*^*R172H/+*^*; Pdx1-Cre*)^15^ mouse model—which combines an activating mutation in *Kras*, and a dominant negative mutation in *Trp53*—is one of the most-studied GEMMs of PDA and has been shown to closely recapitulate the biology of human PDA in terms of histopathological and clinical features^8,15,16^. The *KPTC* (*LSL-Kras*^*G12D/+*^*; LSL-Trp53*^*R172H/+*^*; Tgfbr2*^*flox/+*^*; Ptf1a-Cre*) model is a variation of the *KPC* model that includes a loss of one *Tgfbr2* allele. Features of this model include accelerated carcinogenesis and a lower rate of distant metastasis than the *KPC* model, thus showing promise in phenocopying a subset of PDA patients with oligometastatic disease^17^. The *KPTC* model has overlapping phenotypic features with the *KPDC* model^18^ in which *Smad4* and not *Tgfbr2* is targeted for recombination and inactivation.

While GEMMs of PDA have been extensively studied with respect to biology, detection strategies and therapeutic response^19,20^, their genomic landscape beyond the knock-in transgenic events has remained largely unexplored until recently. One recent study performed whole exome sequencing of four cell lines derived from *KPC* tumors, and found a low mutational burden and lack of antigenic epitopes indicating a lack of immunoediting in this model^21^. A more complete understanding of the frequency and nature of these events is important, as these models are routinely used in preclinical studies of novel therapeutic agents^8,14,22^, and a pervasive assumption is that the tumors are only driven by the *Kras* and *Trp53* transgenes^15^ or that non-cell autonomous factors predominate in driving tumor progression^4^. However, another recent large-scale sequencing study of cell lines from 38 KC (*LSL-Kras*^*G12D/+*^*; Ptf1a-Cre*) mice highlighted the contribution of mutant *Kras* dosage to tumor progression and metastatic potential, thereby providing some of the first evidence of functionally significant genomic events that occur beyond the conditionally activated alleles^23^. To address this important issue, we utilized whole-exome sequencing, aCGH and targeted deep sequencing to extensively characterize the somatic alterations present in a cohort of 12 mice. Moreover, unlike the prior study that used cell lines, we focused on the primary tumor tissues, using a multiregion sequencing strategy, and performed a subclonal phylogenetic reconstruction in each mouse.

While the role of engineered mouse models for understanding PDA genetics has been firmly established, the utility of these models for understanding tumor evolution has not. In this work, we show that in mouse tumors that are induced experimentally, the descendant somatic cancer cells are subject to ongoing evolution and accumulation of somatic alterations. This will pave the way for understanding how different transgenes, modalities of treatment or microenvironmental factors affect tumor evolution and genome chaos, as these and other variables can easily be controlled for in murine models yet are impossible in human patients.

## Results

### Somatic alterations are common in mouse PDA

**Fig. 1:**
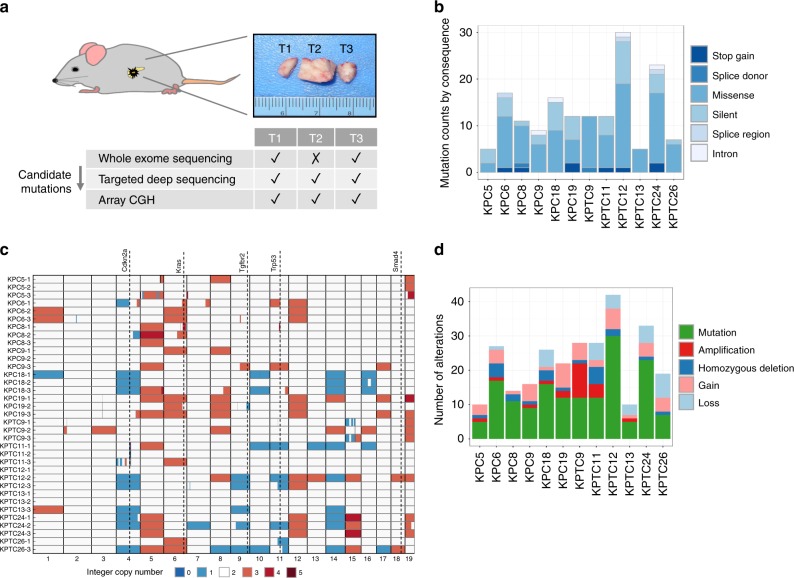
Genomic characterization of six *KPC* and six *KPTC* mice. **a** The tumor tissue collected from the pancreata of each moribund mouse was divided into three regions designated T1–T3. The DNA from tumor regions T1 and T3 was subject to whole exome sequencing. Candidate somatic mutations identified by whole exome sequencing were subsequently validated in all three regions, using targeted amplicon sequencing. Array CGH was applied to the DNA from the three tumor regions. **b** The frequency of mutations with respect to predicted consequence type in each mouse. Values represent those of all three samples per mouse tumor. **c** Somatic copy number profile. The tumor-to-normal copy ratios measured by array CGH were scaled to correct for tumor purity and rounded up to closest integer level. Gain of chr 5, 6, 8, 12, 19 and loss of chr 4, 9, 11, 14 were the most frequent somatic copy number events. **d** The frequency of mutations, focal amplifications and deletions, and large-scale chromosomal gains and losses. Focal and large-scale copy number aberrations were compared across tumor regions from each mouse to identify unique events.

To capture both clonal and subclonal alterations, each tumor harvested from the pancreas of each mouse was divided into three distinct tumor regions in sequential order T1 (left), T2 (middle), and T3 (right) (Fig. 1a). The two non-adjacent regions of the tumor (samples T1 and T3) and a matched normal sample from the kidney from each mouse were profiled by whole exome sequencing to a median sequence coverage depth of 133 × (tumor) and 73 × (normal), identifying somatic alterations in twelve transgenic mice (six *KPC* and six *KPTC)* (Supplementary Datas 1 and 2). Multiregion sequencing and copy number analyses of the tumor tissue from each mouse were applied to enable discovery of subclonal alterations at greater resolution than would be possible with single bulk tissue whole-exome sequencing.

We observed an average of 13(±7) mutations per mouse (0.27 Mut/Mb) (Supplementary Data 3). This estimate was lower than the reported rate in human PDA (~1.0 Mut/Mb^24,25^). However, the trend is consistent with reports from two recent studies of *KP/KPC* cell lines^21,23^ and in an unpublished study of *KPC* mice (described in ref. ^2^). The lower mutation rate could reflect the small number of stem cell divisions that occur in the short life span of *KC/KPC* mice, a lower mutation rate due to lack of exposure to mutagens in a controlled environment, or be a result of unknown differences in the rate of division between mouse and human stem cells. Of a total 159 validated mutations (Fig. 1b), 116 were nonsynonymous (103 missense, 5 at/in proximity of a splice site, and 8 nonsense). No recurrent mutations were observed across the cohort, similar to findings by Mueller et al.^23^. Single nucleotide variant frequencies (e.g., A- > T, C- > G, etc.) were similar in *KPC* and *KPTC* mice (*χ*^2^ test, Benjamini–Hochberg (BH) corrected *p* value > 0.10). Comparison of single nucleotide variant frequencies in our cohort with human PDA data^25,26^ was non-informative, as we detected the presence of batch effects (*χ*^2^ test, BH-corrected *p* value < 0.05) between the available human PDA studies; rendering their observed differences with our data difficult to interpret (Supplementary Fig. 1).

### Somatic copy number aberrations are frequent in mouse PDA

Analysis of somatic copy number aberrations (SCNA) by array CGH of tumor regions in each mouse revealed a substantial degree of aneuploidy, consistent with the presence of a *Trp53* mutation in both *KPC* and *KPTC* genotypes^15^. On average 32 ± 13% of the genes in each mouse were impacted by copy gains/losses, with no significant difference between *KPC* and *KPTC* genotypes (*KPC*: 26 ± 10%, *KPTC*: 38 ± 14%, two-sided *t* test, *p* value: 0.09). The most frequent targets of large-scale copy gain or losses were chromosomes 5, 6, 8, 12, 19 (gains) and chromosomes 4, 9, 11, and 14 (losses) (Fig. 1c, Supplementary Data 4). These chromosomes harbored mouse orthologs of many key genes implicated in human PDA: *Kras* oncogene (chr6), tumor suppressor genes *Cdkn2a* (chr4), *Tgfbr2* (chr9), and *Trp53* (chr11). Notably these gains and losses have also been observed in *Kras-*driven *GEMMs* of lung cancer^27^. There was no significant difference in copy number gains between the *KPC* and *KPTC* genotypes (Fisher’s exact test, two-sided *p* value > 0.05). Copy number losses on chromosomes 9 and 11 occurred only in *KPTC* mice (Fisher’s exact test, two-sided *p* value: 0.06)—a borderline significant result, likely due to our small sample size. Complementary to the array data, an analysis of minor allele frequency of germline heterozygous variants (Supplementary Fig. 2) revealed an enrichment for allelic imbalance in proximity of *Trp53, Kras*, and *Tgfbr2* (Supplementary Fig. 3, Supplementary Data 5).

**Fig. 2:**
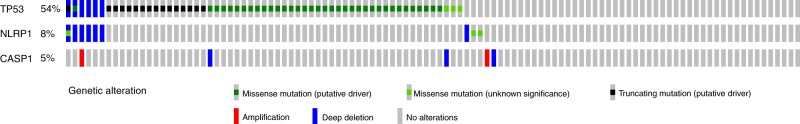
Oncoprint of somatic alterations in TP53, NLRP1, and CASP1 in 109 microdissected human pancreatic cancers (original data from Witkiewicz et al. Nature Communications 2015). Black arrowhead indicates a tumor with *NLRP1* deletion that is *TP53* wild type. Open arrowheads indicate tumors with *CASP1* deletion that are mutually exclusive with *NLRP1* deletions.

Genomic segments harboring homozygous deletion or a gain of at least three copies and less than 5 Mb in size were labeled as focal somatic copy number aberrations (SCNAs). A total of 43 focal SCNAs containing 137 genes were identified (Fig. 1d), with the vast majority of SCNAs (39 of 43, 91%) containing 5 genes or fewer. Genes residing in these regions were screened to determine candidate targets of deletions and amplifications (Supplementary Data 6). Three mice harbored focal amplifications and deletions in well-characterized tumor suppressor genes (*Cdkn2a/b*) and oncogenes (*Cdk6, Myc*) (Supplementary Data 6). We also identified one statistically significant region of recurrent alteration present in 9 of 12 mice (75%), corresponding to a focal deletion on chr11.B4 (70.9–71.1 Mb) containing two paralogs of human *NLRP1*—*Nlrp1b* and *Nlrp1c*^28^ (Supplementary Fig. 4a, Supplementary Data 7, Methods). *NLRP1* is a member of a family of pattern recognition receptors that are critical in mediating inflammation and gastrointestinal defense by innate immunity^29–31^. In all but one mouse this deletion was private to a single region of the tumor indicating it was a subclonal event (Supplementary Fig. 4b). Focal homozygous deletion of *Nlrp1b*, but not the genes immediately upstream or downstream, was confirmed in cell lines derived from three mice (*KPC8*, *KPC9*, and *KPTC26*) in which it was inferred from genomic analyses (Supplementary Fig. 5a, b). In a fourth mouse (*KPTC13*), *Nlrp1b* was predicted to be focally amplified in sample T2 yet the gene could not be detected by quantitayive polymerase chain reaction (qPCR). In a fifth mouse (*KPC6*), *Nlrp1b* was inferred to be focally deleted in sample T1 but was detected in the low passage cell line generated from this tumor, in this case suggesting the cell line was derived from a different subclonal population (see following section for further support of this interpretation). Irrespectively, none of the five cell lines expressed Nlrp1b (Supplementary Fig. 5c). We also determined that the human ortholog *NLRP1* is somatically altered in human pancreatic cancers by screening publicly available whole exome or whole genome sequencing data^26,32,33^. We observed that *NLRP1* is a target of focal homozygous deletion in 6% of a previously documented set of 109 microdissected pancreatic cancers^26^ (Fig. 2). In six patients *NLRP1* deletion was associated with concurrent deletion of *TP53*, also located on chromosome 17p. However, in one patient *NLRP1* was deleted independently, and in three additional patients *CASP1* deletions were identified that were mutually exclusive with *NLRP1* deletions. Thus, our finding of *Nlrp1b* deletions in mice appear to reflect a biologically relevant event in human pancreatic cancer that has not been previously recognized.

### Somatic alterations and critical cellular processes in PDA

While *Kras* and *Trp53* transgene mutations are sufficient to induce PDA with high penetrance, a subset of the inactivating somatic mutations (2 of 10, 20%) and focal SCNAs (6 of 43, 14%) in *KPC* and *KPTC* mice were predicted to have a functional impact on mouse PDA biology by affecting genes previously implicated in key cellular processes^25,34^ (Supplementary Fig. 6, Supplementary Datas 8 and 9). Included in this subset were focal amplifications and deletions, mutations at splice site and nonsense mutations. *KPTC* mice harbored more somatic alterations of all kinds than *KPC*, but the difference was not statistically significant (Fisher’s exact test, *p* > 0.05). Three mice (1 *KPC*, 2 *KPTC*) had mutations or amplification/deletion in genes involved in various stages of DNA damage response; from recognition (splice donor mutation in *Msh3*), to cell cycle control (nonsense mutation in *Trp53*, amplification of *Myc*), and to cellular recovery after DNA damage (Mastl amplification). One *KPTC* mouse harbored amplification of *Acer2*, potentially impacting cell adhesion and the integrin signaling pathway^35^. G1 to S phase transition was impacted in one *KPC* and two *KPTC* mice. In all three, at least one functional focal SCNA was present (*Cdk6* amplification in *KPC5*, *Myc* amplification in *KPTC9*, and *Cdkn2a/b* deletion and *Jun* amplification in *KPTC11*). *KPTC11* also had two *Trp53* tetramerization domain mutations (p.K316N and p.K318*) *in cis* with the engineered mutant allele in the DNA binding domain p.R172H. Furthermore, this mouse, which harbored pancreas-specific loss of a *Tgfbr2* copy, had homozygous loss of *Cdkn2*b, which is an effector of cell cycle arrest by the TGF-beta pathway^36^. The Wnt/Notch signaling pathway was affected in two mice by focal copy number alterations (amplification of *Cdk14* and *Fzd1* in *KPC5* and a deletion of *Mllt3* in *KPTC11*).

Collectively, these findings indicate that the genomic instability in *KPC* and *KPTC* transgenic mice may impact known PDA cancer genes, most often by copy number alterations and to a lesser extent by single-nucleotide substitutions.

### Mouse tumors show evidence of ongoing subclonal evolution

**Fig. 3:**
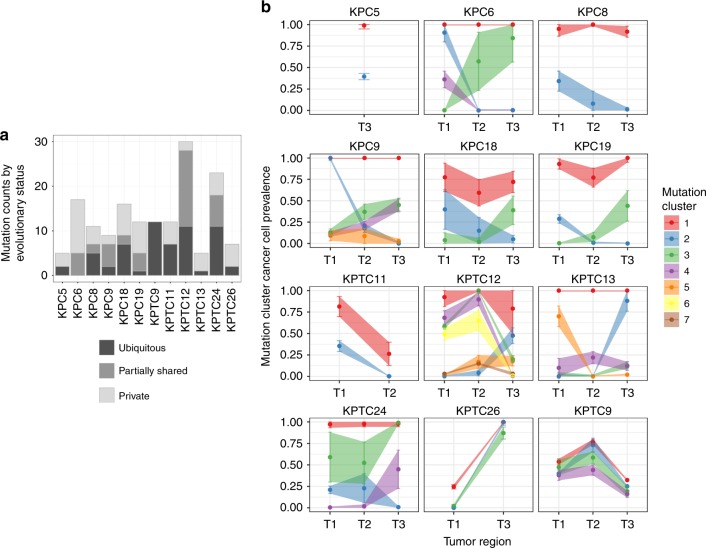
Spatial heterogeneity of somatic mutations across tumor regions. **a** Evolutionary status of somatic mutations in each mouse PDA. Ubiquitous mutations were detected in all analyzed tumor samples of each mouse. Partially shared mutations were present in more than one, but not all, tumor samples. Private mutations were unique to one of the three tumor samples. **b** Mutation cluster cancer cell prevalence distributions across tumor regions. Error bars represent one standard deviation above and below the mean cluster cancer cell prevalence. The prevalence of mutations in each tumor region was estimated by utilizing allele specific read counts from targeted sequencing, somatic copy number, and tumor purity. Mutations were grouped into distinct clusters of similar cancer cell prevalence across tumor regions. Tumor regions T1 and T2 from *KPC5*, T3 from *KPTC11*, and T2 from *KPTC26* were excluded from analysis, due to low variant allele frequency (≦0.02) of all mutations, or insufficient DNA to perform array-CGH (*KPTC26*-T2).

Comparative analysis of the somatic alterations across the three tumor regions from each mouse was used to quantify the extent of spatial heterogeneity and reconstruct the evolutionary history of each PDA. To assess spatial heterogeneity of mutations, we followed the convention used for human solid tumors and categorized them as ubiquitous, partially shared and private^37^, excluding the initiating knock-in transgene mutations. The fraction of ubiquitous, shared and private mutations varied widely across the mice (Fig. 3a), which could be the result of different evolutionary trajectories. In one mouse (*KPC6*), there were no ubiquitous mutations nor mutations shared by tumor regions T1 and T3, indicating the presence of two independent primary tumors that collided in region T2. The transgenic genotype (*KPC* vs. *KPTC*) was not associated with the extent of spatial heterogeneity of mutations (*t* test, two-sided *p* value: 0.47).

**Fig. 4:**
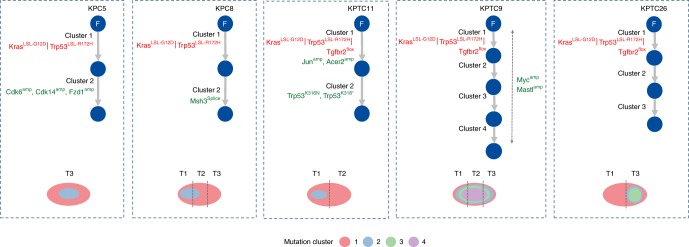
Linear tumor phylogeny in *KPC/KPTC* mice. Mutation cluster cancer cell prevalence was compatible with a linear evolutionary pattern in five mice based on SCHISM analysis, which reconstructs tumor phylogeny as a hierarchy of mutations acquired by cancer cells during the process of tumorigenesis. The schematic diagram beneath each tumor phylogeny depicts the hierarchical order of subclones identified.

**Fig. 5:**
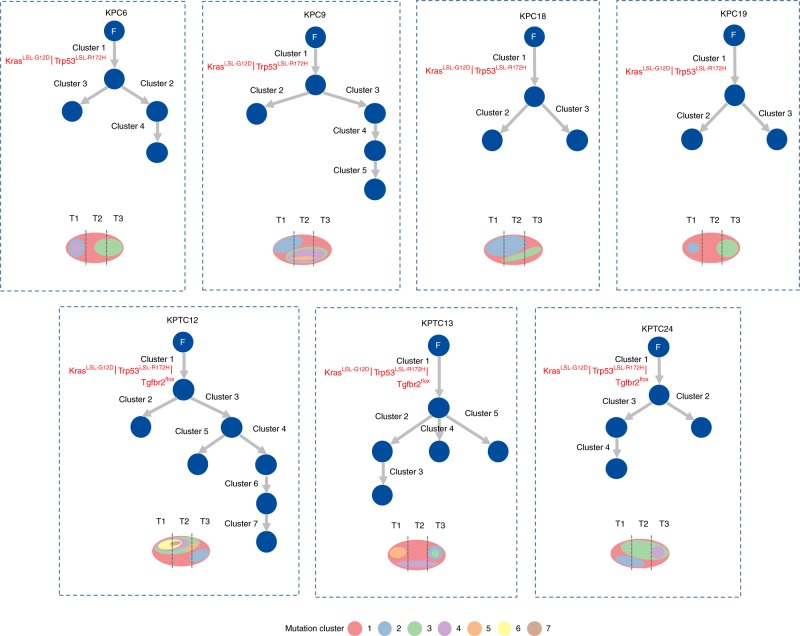
Branched tumor phylogeny in *KPC/KPTC* mice. Seven mice had mutation cluster cancer cell prevalence values compatible with a branched evolutionary pattern. For mice *KPC6*, *KPC9*, *KPTC13*, no mutation cluster appeared at high frequency in all three tumor regions, suggesting two synchronous primary tumors arose in these mice. A mutation cluster consisting of the knock-in tumor initiating mutations was added manually to represent the common ancestral cell. Similar to Fig. 4, a schematic diagram summarizing hierarchical order of subclones is shown for each case.

Next, we modeled the ordering of mutations across tumor regions, based on the estimated proportion of cancer cells harboring each mutation or its *cancer cell prevalence*. Under the infinite sites assumption, commonly used to make this modeling problem tractable^38^, ancestral mutations are expected to have higher cancer cell prevalence than their descendants. Prevalence was estimated by considering a mutation’s variant allele frequency, copy number of its genomic position, and tumor purity (Methods, Supplementary Data 10). Clustering mutations based on cancer cell prevalence, across the three regions of each tumor, yielded distinct mutation groups, which were hypothesized to have appeared at a similar evolutionary time point and to co-localize in the same cells. Two to seven mutation groups were observed in each mouse, with no apparent association between genotype (*KPC* or *KPTC*) and number of groups. Mutation groups with the maximum possible cancer cell prevalence across the three regions as well as those at intermediate to very low prevalence were also identified (Fig. 3b). Intermediate to very low prevalence groups serve as markers of subclones, i.e., cellular populations sharing the same mutations, some of which were not present in the initiating clonal expansion driving the tumor. The existence of these groups supports the presence of subclonal evolution subsequent to the original transgenic events in these mouse tumors. We reconstructed the tumor phylogeny of each mouse tumor by Subclonal Hierarchy Inference from Somatic Mutations (SCHISM) analysis^39^, and observed a similar frequency of linear (5) and branched (7) evolutionary patterns (Figs. 4 and 5). Both patterns occurred in *KPC* and *KPTC* mice. Mice with linear phylogenies had a shorter survival (*t* test, two-sided *p* value: 0.04). Four of five mice with linear phylogenies had amplifications of known oncogenes or somatic mutations in known tumor suppressor genes that likely drove the sequential clonal expansions of the neoplasm (Fig. 4). We noted *Cdk6*, *Ckd14*, and *Fzd1* amplifications in *KPC5*, a subclonal splice site mutation in *Msh3* in *KPC8*, *Jun* and *Acer2* amplifications followed by two additional *Trp53* mutations in *KPTC11*, and *Myc* and *Mastl* amplifications in association with mutations in *Smo* and then *Bcl9* in *KPTC9*. In the case of *KPTC9*, all 12 somatic mutations were present in all three tumor regions, suggesting the *Smo* and *Bcl9* mutations occurred before subclonal sweep caused by the *Myc* amplification (up to 9-fold increased). In *KPTC26* linear progression was also supported by the step-wise accumulation of somatic mutations although no specific gene could be implicated as driving the clonal expansion. In mice with branched phylogenies (Fig. 5), the divergence of tumor lineages occurred either directly after *Cre-lox* mediated activation of the mutant alleles, again consistent with the finding of independent synchronous primary tumors in *KPC6*, or after the accumulation of additional somatic alterations as seen in *KPTC12* and *KPTC24*.

## Discussion

The *KPC* mouse model^15^ is among the most commonly used model for studying PDA due to its faithful recapitulation of the neoplastic histology, desmoplastic stroma and aggressive metastatic spread to regional and distant organs^2,4,8^. We now show that this model and the *KPTC* derivative accumulate additional subclonal somatic alterations, predominantly in the form of copy number alterations that affect core PDA pathways^25^, including those targeted by the transgenes themselves (*Kras*, *Trp53*). Evidence of clonal heterogeneity in engineered mouse PDAs has been previously reported, based on studies of diverse protein expression patterns^15^ and Confetti fluorescent labeling in *KPCX* mice^40^. However, clonal heterogeneity in these models has not previously been observed at the genetic level. Moreover, initial reports of *KPC* genetics indicated a lack of mutations in PDA genes beyond those included in the transgenic constructs^15^ and unbiased multiregion whole exome sequencing of mouse PDA tissues has never been performed. Overall our findings are consistent with those of McFadden et al.^27^ in which additional somatic alterations that converge on the initial oncogenic signal(s) are the most relevant genetic events driving tumor progression in murine models. By contrast, our analysis did not identify any instance of chromothripsis affecting the *Cdkn2a* locus on chromosome 4^23^, likely explained by the limited power for detecting low frequency events in this cohort size and our experimental design. Only one of 12 mice in this study had a homozygous deletion of *Cdkn2a* (*KPTC11*). Consistent with a previous study highlighting the contingency of *Trp53* or *Cdkn2a* alterations in mouse PDA, subsequent to KRAS mutation^23^, the lack of *Cdkn2a* alterations is not surprising.

Because *KPC* PDAs are initiated at the same time, develop within the same genetic background, and in the same environment, they provide unique opportunities to study the evolution of PDA not otherwise possible in patients. Multiregion sequencing of these mouse model tumors makes it feasible to model clonal and subclonal evolution as it occurs in vivo, in an otherwise controlled setting. While our sample size is small in this study, we can already draw some conclusions about PDA evolution in transgenic models. First, these data suggest that evolutionary trajectories arising in the same setting can undergo linear or branched evolution with similar frequencies. Second, PDAs that progress due to linear evolution are associated with shorter lifespans than mice whose PDA follows a branched evolution pattern. This raises interesting follow-up questions regarding the extent to which the microenvironment or immune system pose differences in selective pressure^41^, or the extent to which PDAs progress by way of a genome-based cancer evolution model^42^. Finally, perturbation of a major signaling pathway known to play a role in PDA biology (TGFβ) in *KPTC* (but not *KPC*) mice, does not appear to affect the evolutionary trajectories or mutational frequencies of the mouse tumors in a meaningful way. A limitation of our study is that for those tumors where we sequenced fewer than three regions, our ability to detect subclones and branched evolution is lower.

While gene discovery was not the purpose of this study we nonetheless identified a novel homozygous deletion with potential relevance to human pancreatic cancer. Homozygous deletion of *Nlrp1b* was found in 75% of mice studied, whereas in humans *NLRP1 or its* downstream target *CASP1* is deleted in 9% of cancers analyzed. We note that *NLRP1* deletion was not found in other large scale sequencing studies of pancreatic cancer, potentially because they utilized purified but not microdissected materials for study^32,33^ or the sample size was not powered to detect low frequency events^25^. NLRP1 is a cytosolic sensor of microbial infection that leads to activation of the pro-inflammatory protease CASPASE-1; in turn, CASPASE-1 activation leads to processing and maturation of IL-1β, IL-18, and pyroptosis^43^. Thus, *NLRP1* deletion in mice and humans links perturbation of the innate immune system, and by extension the microbiome, to pancreatic carcinogenesis and progression^44^. Consistent with this notion, Pushalkar et al.^45^ recently illustrated the role of the pancreatic cancer microbiome in promoting oncogenesis by induction of both innate and adaptive immune mechanisms. Furthermore, Daley et al.^46^ indicated a role for NLRP3 signaling in macrophages that leads to a tumor promoting immune environment. Collectively, our data not only suggest that disruption of the NLRP1 inflammasome plays a role in pancreatic cancer progression, but also indicate that disruption may occur by genetic mechanisms. Future functional studies would be needed to confirm this possibility.

## Methods

### Mouse model generation and tissue collection

To generate 6 KPC (LSL-KRASG12D/+;LSL-Trp53R172H/+; Ptf1aCre/+) and 6 KPTC (LSL-KRASG12D/+; LSL-Trp53R172H/+;Tgfbr2flox/+; Ptf1aCre/+) transgenic mice (as previously described 15, 17), the LSL-K-ras G12D, LSL-Trp53R172H/+(B6;129S4-Trp53tm2Tyj/J), Tgfbr2flox/+(B6.129S6-Tgfbr2tm1Hlm) and Ptf1aCre/+(Ptf1atm1.1(cre)Cvw) mouse strains were used. These strains were first bred to generate the genotypes, LSL-KRASG12D/+;Trp53R172H/+(KP) and Tgfbr2flox/+;Ptf1aCre/+(TC), and then the KP mice were intercrossed with TC mice to produce the experimental cohorts, LSL-KRASG12D/+;LSL-Trp53R172H/+; Ptf1aCre/+(KPC) and LSL-KRASG12D/+; LSL-Trp53R172H/+;Tgfbr2flox/+; Ptf1aCre/+(KPTC)^15,17^. On average, the *KPC/KPTC* animals succumbed around 155 ± 52 days (Supplementary Data 1), with no significant difference in survival across the two genotype groups *KPC* (171 ± 66) and *KPTC* (139 ± 30), likely due to our small sample size^17^. In each mouse, recombination was confirmed by examining the rearranged mutant allele(s) in the pancreas and matched normal kidney. The pancreatic tumor tissue harvested from each mouse was grossly macrodissected from surrounding normal tissues and divided into three adjacent regions before immediately snap freezing in liquid nitrogen (Fig. 1a). Histologic review of the non-adjacent tumor regions in each mouse revealed a wide range of differentiation across the cohort (Supplementary Data 1).

All relevant ethical regulations for animal testing and research have been complied with. The study received ethical approval by the Johns Hopkins Animal Care and Use Committee.

### Cell Lines

The KPC and KPTC cell lines were established from PDA tumor tissues derived from KPC and KPTC mice^17^. All lines were cultured in DMEM (GIBCO, Invitrogen Life Technologies, Carlsbad, CA, USA) supplemented with 10% fetal bovine serum (FBS), 100 units/ml penicillin, 100 μg/ml streptomycin, and 2 mmol/L l-glutamine at 37 °C and 5% CO_2_. All lines were also confirmed mycoplasma free before use in any experiments.

### DNA extraction and quality control

Genomic DNA was extracted from all three regions of each *KPC* (*n* = 6) and *KPTC* (*n* = 6) tumor using a DNeasy Blood & Tissue Kit (Cat No./ID 69506; Qiagen). Germline DNA was obtained from matched normal kidney tissue. Extracted DNA was quantified by Nanodrop and Qubit using standard procedures and the integrity of the genomic DNA was confirmed by gel electrophoresis.

### Whole-exome sequencing

Whole-exome sequencing libraries were prepared from the genomic DNA isolated from the non-adjacent pieces T1 and T3 and for matched normal kidney in each mouse, using the Agilent SureSelect^XT^ Mouse All Exons capture kit, per the manufacturer’s protocol. Paired-end whole exome sequencing was performed on an Illumina HiSeq 2500 platform. Preprocessing of sequencing reads from all samples, including de-multiplexing, masking of adaptor sequences, and alignment to mouse reference assembly GRCm38/mm10 was performed in Illumina’s CASAVA v1.8.2^47^, yielding a median sequence coverage depth of 133 × (tumor) and 73 × (normal), and a minimum coverage depth of 10× in 93 ± 5% (tumor) and 86 ± 7% (normal) of bases in the exome target region. Sequence alignment (BAM) files were processed according to the Genome Analysis Toolkit (GATK) best practices guideline^48,49^. Duplicate reads were marked and removed using Picard (v1.11, http://broadinstitute.github.io/picard). Genome Analysis Toolkit (GATK v3.1.1-g07a4bf8) was used to perform local realignment in the proximity of candidate positions with insertion/deletion (indels) and around the set of known polymorphic loci, with indels retrieved from the online catalog of the Mouse Genome Project (v4.0)^50,51^. Base quality scores were recalibrated by GATK, masking out the above set of known polymorphic positions.

### Somatic mutation calling, annotation, and prioritization

Somatic mutations in each tumor sample were identified by processing the matched normal and the tumor reads with MuTect (v1.1.7)^52^, using default parameter settings. The somatic mutations were annotated according to mutation consequence type (missense, nonsense, synonymous, splice site, etc.) with the Ensemble Variant Effect Predictor tool (VEP)^53^. Mutations with differing consequence types across multiple transcripts were assigned the most severe consequence type, according to Ensemble, and Ensemble canonical transcripts were selected when multiple transcripts produced equally severe consequences (http://ensembl.org/info/genome/variation/predicted_data.html). Furthermore, we predicted the pathogenicity of mutations using SIFT^54^, VEST^55^, and REVEL^56^. We limited our analysis to mutations in protein-coding exons with minimum coverage of 10 reads, minimum of 3 variant allele reads and variant allele frequency (VAF) of at least 1%, with at least one read mapped to each of the forward and reverse strands. Mutations were visually inspected in the Integrative Genomics Viewer (IGV v2.3.14)^23,57,58^ to filter out mutation calls in regions of poor alignment quality, regions with low complexity or sequence repeats, or those with more than one variant allele read in normal. A small number of candidate somatic insertions/deletions (indels) were identified in the tumor samples using Scalpel (v0.3.2);^59^ However, these candidate positions did not pass the quality control filters above, and they were mostly located in highly repetitive regions.

To evaluate the relevance of the observed sequence alterations to human pancreatic adenocarcinoma, three tiers of annotation were added to the list of genes harboring somatic mutations. In the first tier, we used our in-house curated database of genes, which are established human PDA drivers or harbor at least two deleterious mutations in five sequencing studies of human PDA^25,26,33,34,60^. The second tier considered the frequency of gene mutation in three sequenced cohorts of human PDA^25,26,34^. Finally, we calculated the number of cases with nonsynymous mutations in 33 TCGA tumor types.

The frequency of gene mutation in three PDA studies was retrieved using Supplementary Data 1 from^26^. TCGA Multi-Center Mutation Calling in Multiple Cancers (MC3) mutation calls were downloaded from synapse (10.7303/syn7214402). The mapping between sample barcodes and tumor type was retrieved using the package TCGAbiolinks-2.2.10^61^ in R version 3.3.2, and the number of cases with nonsynomous mutations across the samples in each tumor type was reported.

### Somatic mutation validation

Targeted amplicon sequencing at candidate somatically mutated positions that passed the above filtering criteria was performed on all three tumor regions of each mouse. Amplicons were sequenced in the MSK Integrated Genomics Operating (IGO) Core using the Ion PGM^TM^ system and aligned to the mouse reference assembly (mm10) using tmap, the manufacturer’s alignment tool. The resulting sequence alignment files were processed using the GATK utility, as with the whole exome data, but omitting the duplicate read removal step. Allele-specific read counts for sequencing reads mapping to forward and reverse strands were generated by samtools^62^. Candidate mutations passing four filters were marked as validated: (1) minimum variant allele frequency of 1% in at least one tumor region; (2) minimum variant read count of five in at least one tumor region; (3) minimum of three reads mapping to the forward strand in at least one tumor region; and (4) minimum of three reads mapping to the reverse strand in at least one tumor region. The stricter filtering criteria, compared to whole-exome sequencing, were motivated by higher sequencing coverage and base error rate in the amplicon resequencing. The set of validated mutations from all mice was visually reviewed in the Integrative Genomics Viewer (IGV)^58^ to confirm the quality of filter performance. The validated set included 159 high confidence mutations, with median sequence coverage of 334 × (MAD = 108). The targeted sequencing allowed us to identify false positive mutation calls, to screen for mutations in tumor region T2, for which whole exome sequence was not available, and to yield higher sequence coverage and more accurate VAF estimates.

### Germline variant identification

An initial set of candidate germline single-nucleotide polymorphisms (SNPs) were identified from the exome data of the matched normal (kidney) sample of each mouse using GATK UnifiedGenotyper^48,49,63^, by setting the standard minimum threshold for calling positions to 30, and down-sampling reads at regions with excessive coverage to 250×. This initial set was filtered to exclude positions with depth of coverage (DP) less than 20, quality score normalized by allelic depth (QD) less than 2, Fisher’s strand bias (FS) greater than 60, mapping quality (MQ) less than 40, haplotype score (HS) greater than 13, MQ rank sum (MQRankSum) less than −12.5, or read position rank sum (ReadPosRankSum) less than −8.0.

### Tumor purity estimation

The fraction of cancer cells in each tumor region (tumor purity) was determined by a custom-designed real-time PCR-based assay. The assay is based on a polymerase chain reaction-amplified sequence of 270 bp of *Trp53* LSL cassette, using the primer pair T036 (5′-agc tag cca cca tgg ctt gag taa gtc tgc a-3′) and T035 (5′-ctt gga gac ata gcc aca ctg-3′). Upon expression of the Cre recombinase enzyme, the LSL cassette is removed in tumor cells, but not in cells from non-cancer tissue. Then the PCR assay can determine the abundance of non-cancer tissue in the *KPC* and *KPTC* tumor samples. To calculate the purity, a standard reference was established by mixing *KPC* pancreatic cancer cell line gDNA with that of non-tumor kidney from *KPC* mice at varying proportions. The standard categories were set up as 0, 10, 20, 40, 60, 80, and 100% of pancreatic cancer cells. Genomic DNA from tumor tissues or standards was quantified by Qubit fluorometer (Invitrogen) and Nanodrop, and 10 ng DNA was subjected to qPCR in a total volume of 20 μl reaction system, using primers targeting *Trp53* LSL cassette. Primer pair (Forward: 5′-GTAGCCATCCAGGCTGTGCTG-3′; Reverse: 5′-GATGGGCACAGTGTGGGTGAC-3′) amplifying 90 bp β-actin genomic sequence was set up as an internal control to normalize the input amount of gDNA for qPCR. Cycle threshold (Ct) values from the standard reference experiment above (performed in triplicate) were used to fit a regression model *Y* = *AX* + *B*, mapping the purity (*X*) to the read out Ct value (*Y*). The fraction of pancreatic cancer cells in each *KPC* or *KPTC* tumor tissue was estimated using the regression model. The assay was repeated a minimum of three times for each sample per mouse, and the average of purity estimates was reported (Supplementary Data 11).

### Array comparative genomic hybridization

Array comparative genomic hybridization (aCGH) was performed by labeling DNA from each tumor region and matched normal sample of each mouse with Cy5 or Cy3 fluorescent dyes. The labeled pool was hybridized to Agilent-014695 Mouse Genome CGH Microarray 244A. The microarray, which is optimized for copy number profiling, includes more than 235,000 distinct biological features, with median spacing of 6.2 and 15.2 kb in coding, and noncoding genomic sequences, respectively. The arrays were scanned and preprocessed by Agilent Feature Extraction software following manufacturer’s protocols. Background subtraction and within array normalization (loess) of intensity values were performed by bioconductor limma package (v3.22.7)^64–66^ in R statistical software (v3.2.3)^67^. Genome regions with constant copy number were identified by segmenting the log ratio of tumor to normal intensities using circular binary segmentation with default parameters implemented in bioconductor DNAcopy package (v1.40.0)^68^ in R statistical software (v3.2.3)^67^. To reduce over-segmentation due to noise in data, splits between segments with mean values within 3 standard deviation of each other were removed by the “undo” method of the same package. Segment log2 ratio values were corrected for variable tumor purity in each region. The observed log2 ratio of tumor to normal signal, in a sample with tumor purity *p* is

1 $${\rm{log}}_2R^{\rm{obs}} = {\rm{log}}_2\left( {\frac{{p{\mathrm{CN}}_T + \left( {1 - p} \right){\mathrm{CN}}_N}}{{{\mathrm{CN}}_N}}} \right),$$

where CN_*T*_ and CN_*N*_ are the integer copy number of the segment in tumor and normal cells. Since tumor purity *p* and normal copy number CN_*N*_ are known, given an observed value of log2 ratio, we can solve for tumor copy number CN_*T*_. CN_*T*_ was rounded to the closest integer. Autosomal regions with with CN_*T*_ = 0 were labeled as homozygous deletions, and those with a minimum CN_*T*_ of 5 were called amplified. Large scale copy gains or losses were defined as alterations spanning at least 50% of the chromosome. Genomic coordinates from NCBI reference sequence collection (RefSeq)^69^ in mm9 coordinates were retrieved, and intersected with copy number segment coordinates to derive the somatic integer copy number of genes in each tumor region. Focal amplifications and deletions were defined as genomic segments with homozygous deletion (HD: CN_*T*_ = 0) or gain of at least three copies $({\mathrm{Amp}}:{\mathrm{CN}}_T \ge 5)$, and less than 4 Mb in size. These segments were narrowed down to those covering at least five array probes and located on autosomes. Focal aberrations of same class (HD or Amp) were merged if their boundaries were less than 100 kb apart. To identify the evolutionary status of focal copy number events, aberrations detected in multiple regions of the same mouse, and sharing an overlap of at least 50% (overlap/union) were considered to be same events. The segments were further filtered to only include those which overlap the coding sequence of at least one gene (RefSeq, mm9) (Table S6).

GISTIC v2.0 was applied with default parameters, and confidence level of 0.9 on a collection of 12 tumor regions (including the tumor region with highest purity from each mouse) to assess the genome wide significance of focal copy number alterations. False-discovery rate (FDR) of 0.05 was used as the significance threshold (Table S7).

### Exome-wide allelic imbalance analysis

By evaluation of minor allele frequencies (MAFs) along the exome, we screened for candidate genes which harbored allelic imbalance (including loss of heterozygosity). Genomic coordinates of genes annotated on reference assembly mm10 were retrieved from UCSC’s refGene table (including protein-coding and non-protein-coding genes from the NCBI RNA reference sequences collection, RefSeq)^69,70^. Allelic imbalance in the region around each gene was determined by comparison of the MAF of ten germline heterozygous SNPs closest to the gene mid-point between tumor and matched normal samples in each mouse as follows. At each position harboring a germline heterozygous SNP, the number of reads with the reference and alternate alleles in each sample were recorded using samtools (v0.1.19)^62,71^. Positions with DP below 20 were labeled as uninformative and excluded from analysis. Because the observed MAF in regions with one copy loss deviated from the expected value of 0 (due to normal contamination), simulation experiments were performed to identify the optimal MAF threshold to call allelic imbalance, taking into account the estimated purity and sequencing coverage in each tumor region. Given the expected value of minor allele frequency in a region with one copy deletion, in a tumor with purity of *p*

2 $${\mathrm{MAF}}^{\rm{tumor}} = \frac{{(1 - p)}}{{(2 - p)}}.$$

We simulated the reference and alternate read counts for a batch of 10,000 hypothetical SNPs in tumor and normal sample (${\mathrm{MAF}}^{\rm{normal}}$ = 0.5), using a binomial process, with the number of trials set to the sample sequencing coverage. MAF values were averaged over subsets of size 10, yielding 1000 data points in tumor and normal. At each threshold level between 0 and 0.50 in increments of 0.01, sensitivity/specificity/F1-score values for classifying allelic imbalance as below the threshold were calculated. In each sample, we determined a threshold yielding a sensitivity and specificity exceeding 0.8. Samples where such threshold could not be found (commonly due to low tumor purity) were labeled as lacking power for allelic imbalance analysis (*KPC5*-T1, *KPC9*-T3, and KPTC9-T3). In the remaining samples, the threshold value with maximum F1-score was select as optimal MAF threshold value of each sample, from the subset of all thresholds satisfying the minimum sensitivity and specificity criteria. The mid-point for each gene was defined as the average between the coding sequence start and end coordinates. The MAF values of 10 closest SNPS to each gene (mid-point) were compared between tumor and matched normal sample using one-tailed *t* test, and the *p* values were corrected for multiple hypothesis testing using Benjamini Hochberg procedure. Genes with mean tumor MAF below the optimized threshold, and with FDR < 5% were labeled as harboring allelic imbalance. In each mouse, the set of genes with allelic imbalance was generated by taking the union of all such genes in tumor regions T1, and T3. Background rates of gene allelic imbalance were derived for the mice with *KPC* genotype, those with *KPTC* genotype, and the entire cohort. In each gene, the enrichment for allelic imbalance was assessed using a binomial process and the background rates above. Benjamini–Hochberg correction was applied to the resulting *p* values.

### Subclonal hierarchy inference and tumor phylogeny reconstruction

SCHISM^39^ was applied to reconstruct the evolutionary history of PDAC in each mouse. For each somatic mutation in each tumor region, allelic read counts were extracted from targeted amplicon sequencing. We combined the reference and variant read counts for each mutation with estimated tumor purity and somatic integer copy number, to construct a point estimate and confidence interval for the cancer cell prevalence, in each tumor region. The expected variant allele frequency of a mutation with cellular prevalence, multiplicity *m*, and in a genomic region with tumor copy number CN_*T*_ and normal copy number CN_*N*_ is

3 $$v_{\rm{exp}} = \frac{{pmC}}{{p{\mathrm{CN}}_T + \left( {1 - p} \right){\mathrm{CN}}_N}},$$

multiplicity $m \in \left[ {1,{\mathrm{CN}}_T} \right]$ is the number of mutation copies present in a tumor cell. The variant read count *r*_*A*_ is modeled as number of successes in a binomial process where the number of trials *r*_tot_ is equal to the DP, and the probability of success is *v*_exp_. Given CN_*T*_, we constructed the confidence interval over *C* for each possible value of *m* by normalizing the binomial probability over a grid of cancer cell prevalence values *p* between 0 and 1, with 0.01 increments. For mutations located in regions with ${\mathrm{CN}}_T \in \left[ {1,2} \right]$ only one value of multiplicity (*m* = 1) is plausible. The prevalence of mutations in regions with copy gain was treated as a missing value, unless deductive reasoning could rule out all but one multiplicity value. Examples include cases where certain values of *m* resulted in cellular prevalence exceeding 1, or cases where the mutation was absent in a tumor region and the point estimate of cellular prevalence was at 0.0 regardless of value of *m* (Table S9). A statistical hypothesis test was applied to assess the potential temporal ordering of each pair of mutations. Clustering mutations based on similar cancer cell prevalence across tumor regions yielded mutation groups expected to have originated at the same evolutionary time point and to occur in the same cells. Clustering was performed with DBSCAN^72^ (Scikit-learn v0.14.1^73^, and the number of mutation groups was selected by comparison of silhouette coefficients, and the final solution was visually examined for quality control (Fig. 3b). In three mice (*KPC9*, *KPTC12*, *KPTC13*) where DBSCAN was unable to find high quality solutions, and affinity propagation^74^ (Scikit-learn v0.14.0) was applied as a secondary clustering approach. SCHISM v1.1.1 was run with default parameter settings to yield the phylogenetic trees representing tumor evolutionary histories (Figs. 4 and 5). Tumor regions T1 and T2 from *KPC5*, T3 from *KPTC11*, and T2 from *KPTC26* were excluded from SCHISM analysis due to low variant allele frequency (≦0.02) across all mutations, or unavailability of a copy number profile (*KPTC26*-T2).

### Cell lines

The establishment of *KPC* and *KPTC* cell lines has previously been described^17^. Briefly, cell lines were passaged at least five times to remove all non-neoplastic elements, then genotyped by PCR to confirm they contain the recombined alleles. Cells were implanted both subcutaneously and orthotopically to confirm tumorigenicity in CD1^nu/nu^ mice. All lines were cultured in DMEM (GIBCO, Invitrogen Life Technologies, Carlsbad, CA, USA) supplemented with 10% FBS, 100 units/ml penicillin, 100 μg/ml streptomycin, and 2 mmol/L l-glutamine at 37 °C and 5% CO_2_. All lines were also confirmed mycoplasma free before use in any experiments.

### qPCR for validating a deletion on Chr11.B4 (70.9–71.1 Mb)

Genomic DNA (gDNA) were extracted from cells using a DNeasy Blood & Tissue Kit (Cat No./ID 69506; Qiagen). gDNA obtained from tumor-free kidney tissue were used as wild-type control of chr11: 70.9–71.1 Mb locus. Extracted DNA was quantified by Nanodrop and Qubit using standard procedures, and 10 ng of gDNA was subjected to qPCR analysis. The following primer pair amplifying a 190 bp fragment located at the up-stream of Nlrp1b gene within the deleted locus was used for this assay: forward, 5′-ggagatcgcatagctcagttg-3′ (CHR_CAST_EI_MMCHR11_CTG4: 71,065,735-71,065,757); reverse, 5′-gtgtccaacagcccagaaata-3′ (CHR_CAST_EI_MMCHR11_CTG4: 71,065,904-71,065,926). Primer pairs targeted against three different genes flanked 70.9–71.1 Mb region were also included into the qPCR assay: Forward 5′-gatgtggcgc cacagctgct c-3′ and reverse 5′-ctggtccttctctctgcgttg-3′ for Derl2; Forward 5′-ccacagactt gcctgctgag g-3′ and reverse 5′-ctgctcctctaattctgcaag-3′ for Mis12; Forward 5′-ctagagct gtgctaccga agc-3′ and reverse 5′-cagggcctcccatatgagattc-3′ for Nlrp1b-1c-ps. Primer pair (Forward: 5′-gtagccatccaggctgtgctg-3′; Reverse: 5′-gatgggcacagtgtgggtgac-3′) amplifying 90 bp β-actin genomic sequence was set up as an internal control to normalize the input amount of gDNA for qPCR. Real-time quantitative PCR analysis was performed using an automated sequence detection instrument (7300 Real Time PCR System, Applied Biosystems, CA, USA) for the real-time monitoring of nucleic acid green dye fluorescence (SYBR®Green, Invitrogen Inc, CA, USA). Relative fold-changes of the target locus compared to β-actin locus were determined by calculation of the 2^ΔΔCt^. All analyses were performed in triplicate at least two times.

### Real time qRT-PCR

Total RNA was extracted from *KPC* and *KPTC* cells using RNeasy mini Kit (Cat. No. 74104; Qiagen). RNA was treated with DNase I (Invitrogen) to digest remnant genomic DNA. cDNA was synthesized from 0.5 μg of total RNA by the High-Capacity cDNA Reverse Transcription Kits (Applied Biosystems) according to the protocol recommended by the manufacturer. Real-time quantitative RT-PCR analysis was performed using an automated sequence detection instrument (7300 Real Time PCR System, Applied Biosystems, CA, USA) for the real-time monitoring of nucleic acid green dye fluorescence (SYBR^®^Green, Invitrogen Inc., CA, USA). Relative fold-changes of analyzed gene expression compared to the housekeeping gene β-actin were determined by calculation of the 2^ΔΔCt^. All analyses were performed in triplicate at least two times. Primer sequences will be provided upon request.

### Reporting summary

Further information on research design is available in the Nature Research Reporting Summary linked to this article.

## Supplementary information

Supplementary Information

Description of Additional Supplementary Files

Supplementary Data 1

Supplementary Data 2

Supplementary Data 3

Supplementary Data 4

Supplementary Data 5

Supplementary Data 6

Supplementary Data 7

Supplementary Data 8

Supplementary Data 9

Supplementary Data 10

Supplementary Data 11

Reporting Summary

## Data Availability

Whole-exome and targeted sequence data of the *KPC* (*n* = 6) and *KPTC* (*n* = 6) tumor samples are available on the NCBI Short Read Archive database (Accession # PRJNA546566). Array CGH data of the same sample sets is available on the Gene Expression Omnibus database (Accession # GSE132235). All processed data relevant to this work are available in the Supplementary Files (Supplementary Information, Description of Additional Supplementary Files and Supplementary Datas 1–11). All other relevant data are available upon request.

## Code availability

All bioinformatics methods employed in the analyses are publicly available and referenced under Methods.

## References

[1] Hingorani SR (2003). Preinvasive and invasive ductal pancreatic cancer and its early detection in the mouse. Cancer Cell.

[2] Guerra C, Barbacid M (2013). Genetically engineered mouse models of pancreatic adenocarcinoma. Mol. Oncol..

[3] Ijichi H (2011). Genetically-engineered mouse models for pancreatic cancer: Advances and current limitations. World J. Clin. Oncol..

[4] Perez-Mancera PA, Guerra C, Barbacid M, Tuveson DA (2012). What we have learned about pancreatic cancer from mouse models. Gastroenterology.

[5] De LaOJ (2008). Notch and Kras reprogram pancreatic acinar cells to ductal intraepithelial neoplasia. Proc. Natl Acad. Sci. USA.

[6] Habbe N (2008). Spontaneous induction of murine pancreatic intraepithelial neoplasia (mPanIN) by acinar cell targeting of oncogenic Kras in adult mice. Proc. Natl Acad. Sci. USA.

[7] Beatty GL (2011). CD40 agonists alter tumor stroma and show efficacy against pancreatic carcinoma in mice and humans. Science.

[8] Olive KP (2009). Inhibition of Hedgehog signaling enhances delivery of chemotherapy in a mouse model of pancreatic cancer. Science.

[9] Guerra C (2011). Pancreatitis-induced inflammation contributes to pancreatic cancer by inhibiting oncogene-induced senescence. Cancer Cell.

[10] Lee KE, Bar-Sagi D (2010). Oncogenic KRas suppresses inflammation-associated senescence of pancreatic ductal cells. Cancer Cell.

[11] Guerra C (2007). Chronic pancreatitis is essential for induction of pancreatic ductal adenocarcinoma by K-Ras oncogenes in adult mice. Cancer Cell.

[12] Morris (2010). blocks Kras-dependent reprogramming of acini into pancreatic cancer precursor lesions in mice. J. Clin. Invest..

[13] Faca VM (2008). A mouse to human search for plasma proteome changes associated with pancreatic tumor development. PLoS Med..

[14] Olive KP, Tuveson DA (2006). The use of targeted mouse models for preclinical testing of novel cancer therapeutics. Clin. Cancer Res..

[15] Hingorani SR (2005). Trp53R172H and KrasG12D cooperate to promote chromosomal instability and widely metastatic pancreatic ductal adenocarcinoma in mice. Cancer Cell.

[16] Hruban RH (2006). Pathology of genetically engineered mouse models of pancreatic exocrine cancer: consensus report and recommendations. Cancer Res..

[17] Zhong Y (2017). Mutant p53 together with TGFbeta signaling influence organ-specific hematogenous colonization patterns of pancreatic cancer. Clin. Cancer Res.

[18] Whittle MC (2015). RUNX3 controls a metastatic switch in pancreatic ductal adenocarcinoma. Cell.

[19] Provenzano PP (2012). Enzymatic targeting of the stroma ablates physical barriers to treatment of pancreatic ductal adenocarcinoma. Cancer Cell.

[20] Van Dyke T (2010). Finding the tumor copycat: approximating a human cancer. Nat. Med..

[21] Evans R. A., et al. Lack of immunoediting in murine pancreatic cancer reversed with neoantigen. *JCI Insight* **1** (2016). https://insight.jci.org/articles/view/88328. 10.1172/jci.insight.88328 PMC5026128 27642636

[22] Lee JW, Komar CA, Bengsch F, Graham K, Beatty GL (2016). Genetically Engineered Mouse Models of Pancreatic Cancer: The KPC Model (LSL-Kras(G12D/+);LSL-Trp53(R172H/+);Pdx-1-Cre), its variants, and their application in immuno-oncology drug discovery. Curr. Protoc. Pharm..

[23] Mueller S (2018). Evolutionary routes and KRAS dosage define pancreatic cancer phenotypes. Nature.

[24] Alexandrov LB (2013). Signatures of mutational processes in human cancer. Nature.

[25] Jones S (2008). Core signaling pathways in human pancreatic cancers revealed by global genomic analyses. Science.

[26] Witkiewicz AK (2015). Whole-exome sequencing of pancreatic cancer defines genetic diversity and therapeutic targets. Nat. Commun..

[27] McFadden DG (2016). Mutational landscape of EGFR-, MYC-, and Kras-driven genetically engineered mouse models of lung adenocarcinoma. Proc. Natl Acad. Sci. USA.

[28] Sastalla I (2013). Transcriptional analysis of the three Nlrp1 paralogs in mice. BMC Genomics.

[29] Davis BK (2014). Emerging significance of NLRs in inflammatory bowel disease. Inflamm. Bowel Dis..

[30] Dupaul-Chicoine J (2010). Control of intestinal homeostasis, colitis, and colitis-associated colorectal cancer by the inflammatory caspases. Immunity.

[31] Williams TM (2015). The NLRP1 inflammasome attenuates colitis and colitis-associated tumorigenesis. J. Immunol..

[32] Cancer Genome Atlas Research Network. (2017). Electronic address aadhe, Cancer Genome Atlas Research N. Integrated Genomic Characterization of Pancreatic Ductal Adenocarcinoma. Cancer Cell.

[33] Waddell N (2015). Whole genomes redefine the mutational landscape of pancreatic cancer. Nature.

[34] Biankin AV (2012). Pancreatic cancer genomes reveal aberrations in axon guidance pathway genes. Nature.

[35] Sun W (2009). Alkaline ceramidase 2 regulates beta1 integrin maturation and cell adhesion. FASEB J..

[36] Hannon GJ, Beach D (1994). p15INK4B is a potential effector of TGF-beta-induced cell cycle arrest. Nature.

[37] Gerlinger M (2012). Intratumor heterogeneity and branched evolution revealed by multiregion sequencing. N. Engl. J. Med..

[38] Kimura M (1969). The number of heterozygous nucleotide sites maintained in a finite population due to steady flux of mutations. Genetics.

[39] Niknafs N, Beleva-Guthrie V, Naiman DQ, Karchin R (2015). SubClonal hierarchy inference from somatic mutations: automatic reconstruction of cancer evolutionary trees from multi-region next generation sequencing. PLoS Comput. Biol..

[40] Maddipati R, Stanger BZ (2015). Pancreatic cancer metastases harbor evidence of polyclonality. Cancer Discov..

[41] Makohon-Moore Alvin, Iacobuzio-Donahue Christine A. (2016). Pancreatic cancer biology and genetics from an evolutionary perspective. Nature Reviews Cancer.

[42] Heng Henry H. Q. (2009). The genome-centric concept: resynthesis of evolutionary theory. BioEssays.

[43] Hayward J. A., Mathur A., Ngo C., Man S. M. Cytosolic recognition of microbes and pathogens: inflammasomes in action. *Microbiol. Mol. Biol. Rev.* **82** (2018). https://mmbr.asm.org/content/82/4/e00015-18. 10.1128/MMBR.00015-18 PMC6298609 30209070

[44] Zambirinis CP, Miller G (2017). Cancer manipulation of host physiology: lessons from pancreatic cancer. Trends Mol. Med..

[45] Pushalkar S (2018). The pancreatic cancer microbiome promotes oncogenesis by induction of innate and adaptive immune suppression. Cancer Discov..

[46] Daley D (2017). NLRP3 signaling drives macrophage-induced adaptive immune suppression in pancreatic carcinoma. J. Exp. Med..

[47] Illumina I (2010). CASAVA Software Version 1.7 User Guide..

[48] DePristo MA (2011). A framework for variation discovery and genotyping using next-generation DNA sequencing data. Nat. Genet..

[49] Van der Auwera GA (2013). From FastQ data to high confidence variant calls: the Genome Analysis Toolkit best practices pipeline. Curr. Protoc. Bioinforma..

[50] Keane TM (2011). Mouse genomic variation and its effect on phenotypes and gene regulation. Nature.

[51] Yalcin B, Adams DJ, Flint J, Keane TM (2012). Next-generation sequencing of experimental mouse strains. Mamm. Genome.

[52] Cibulskis K (2013). Sensitive detection of somatic point mutations in impure and heterogeneous cancer samples. Nat. Biotechnol..

[53] McLaren W (2016). The ensembl variant effect predictor. Genome Biol..

[54] Ng PC, Henikoff S (2006). Predicting the effects of amino acid substitutions on protein function. Annu Rev. Genomics Hum. Genet..

[55] Carter H, Douville C, Stenson PD, Cooper DN, Karchin R (2013). Identifying Mendelian disease genes with the variant effect scoring tool. BMC Genomics.

[56] Ioannidis NM (2016). REVEL: an ensemble method for predicting the pathogenicity of rare missense variants. Am. J. Hum. Genet..

[57] Robinson JT (2011). Integrative genomics viewer. Nat. Biotechnol..

[58] Thorvaldsdottir H, Robinson JT, Mesirov JP (2013). Integrative Genomics Viewer (IGV): high-performance genomics data visualization and exploration. Brief. Bioinform..

[59] Narzisi G (2014). Accurate de novo and transmitted indel detection in exome-capture data using microassembly. Nat. Methods.

[60] Bailey P (2016). Genomic analyses identify molecular subtypes of pancreatic cancer. Nature.

[61] Colaprico A (2016). TCGAbiolinks: an R/Bioconductor package for integrative analysis of TCGA data. Nucleic Acids Res..

[62] Li H (2009). The Sequence Alignment/Map format and SAMtools. Bioinformatics.

[63] McKenna A (2010). The Genome Analysis Toolkit: a MapReduce framework for analyzing next-generation DNA sequencing data. Genome Res..

[64] Ritchie ME (2015). limma powers differential expression analyses for RNA-sequencing and microarray studies. Nucleic Acids Res..

[65] Ritchie ME (2007). A comparison of background correction methods for two-colour microarrays. Bioinformatics.

[66] Smyth GK, Speed T (2003). Normalization of cDNA microarray data. Methods.

[67] R Core Team. *R: A Language and Environment for Statistical Computing*. (2014). http://www.R-project.org/.

[68] Venkatraman ES, Olshen AB (2007). A faster circular binary segmentation algorithm for the analysis of array CGH data. Bioinformatics.

[69] Pruitt KD, Tatusova T, Maglott DR (2005). NCBI Reference Sequence (RefSeq): a curated non-redundant sequence database of genomes, transcripts and proteins. Nucleic Acids Res..

[70] Karolchik D (2004). The UCSC Table Browser data retrieval tool. Nucleic Acids Res..

[71] Li H. *Mathematical Notes on SAMtools Algorithms*. (2012). https://software.broadinstitute.org/gatk/media/docs/Samtools.pdf.

[72] Ester M., Kriegel H. P., Sander J., Xu X. A density-based algorithm for discovering clusters in large spatial databases with noise. In: *Proc 2nd International Conference on Knowledge Discovery and Data Mining (*AAAI Press, 1996).

[73] Pedregosa F (2011). Scikit-learn: machine learning in Python. J. Mach. Learn. Res..

[74] Frey BJ, Dueck D (2007). Clustering by passing messages between data points. Science.

