# Supplementary material for: Characterization of genetic subclonal evolution in pancreatic cancer mouse models
Source: Nat Commun. 2019 Nov 28;10:5435. doi: 10.1038/s41467-019-13100-w (PMC6882784; doi:10.1038/s41467-019-13100-w)
Supplement: Supplementary file 1 — Supplementary Information [file 41467_2019_13100_MOESM1_ESM.pdf]

Supplementary Information for

Characterization of Genetic Subclonal Evolution in Pancreatic Cancer Mouse Models

Noushin Niknafs et al.

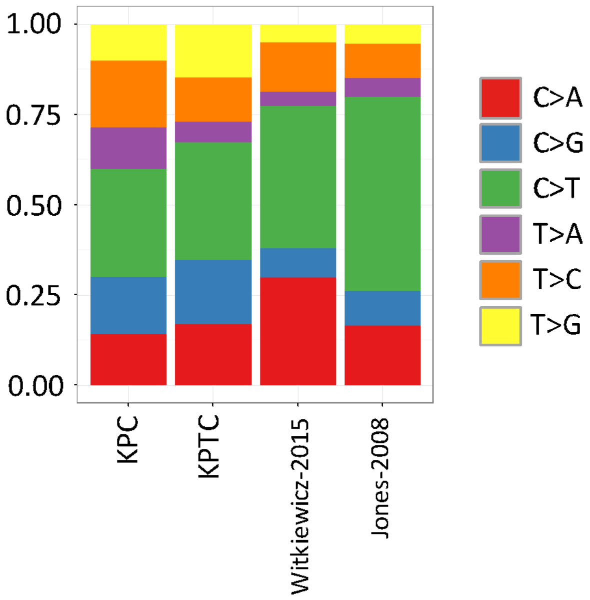

Supplementary Figure 1. Comparison of mutation spectra of the single base substitutions (SBS) identified in this cohort, with previously published studies of human PDA. Related to Figure 1B. The mutation spectra for SBSs from any two human PDA studies/resource exhibited significant statistical difference (χ^2 test of homogeneity, p-value < 0.05), which suggests presence of batch effects and restricts our ability to use them as reference of comparison for the mouse data. No significant difference was observed between the mutations identified in mice with *KPC* and *KPTC* genotype.

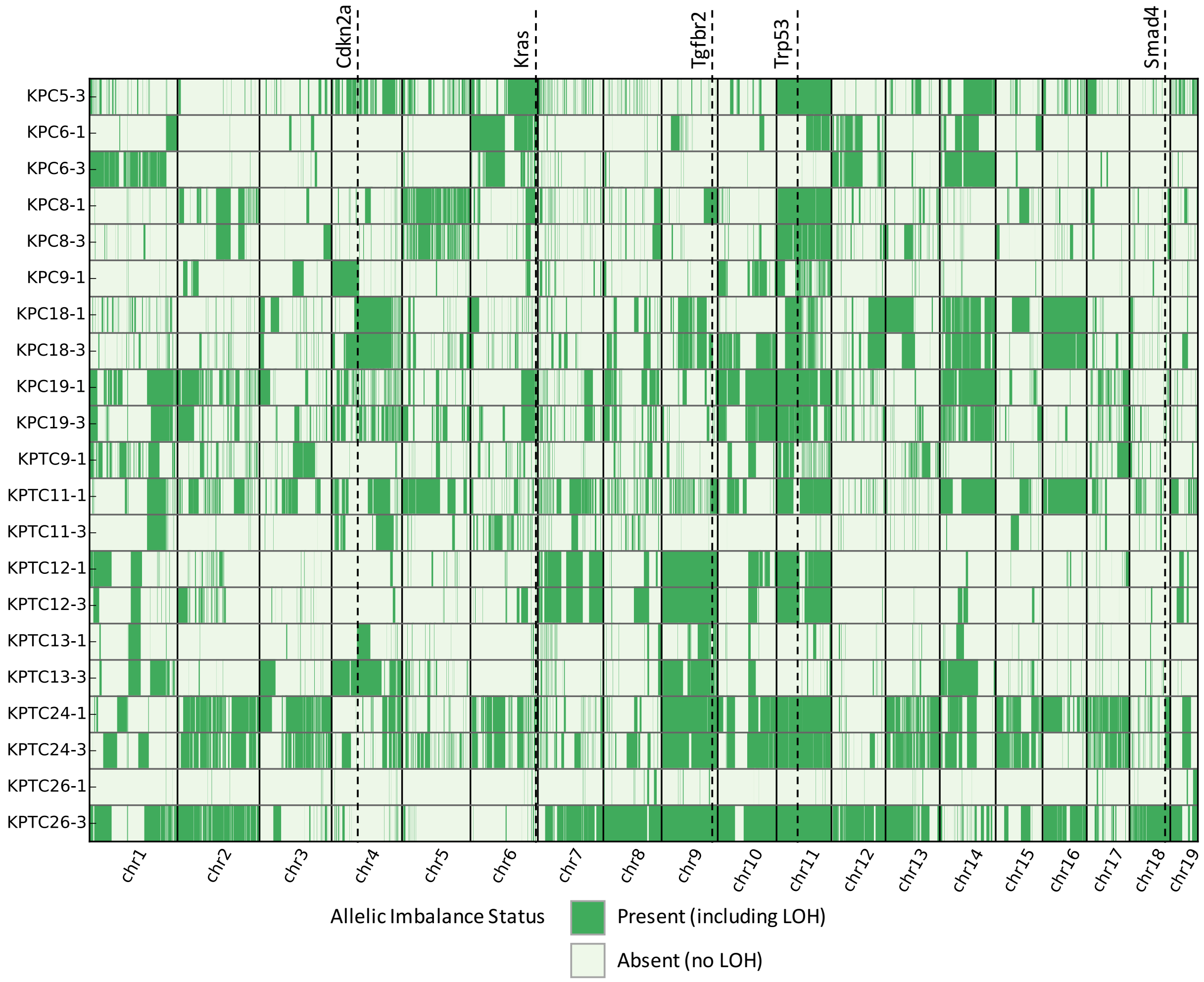

Supplementary Figure 2. Allelic imbalance profile across the genome. Related to Figure 1C. In tumor regions T1 and T3 from each mouse where whole exome data was available, the allelic imbalance (proxy for LOH) status of each gene was evaluated using the minor allele frequency distribution of the 10 closest germline heterozygous SNPs. A comparison was performed between the minor allele frequency of matched normal and tumor sample, taking into account tumor purity and sequencing coverage.

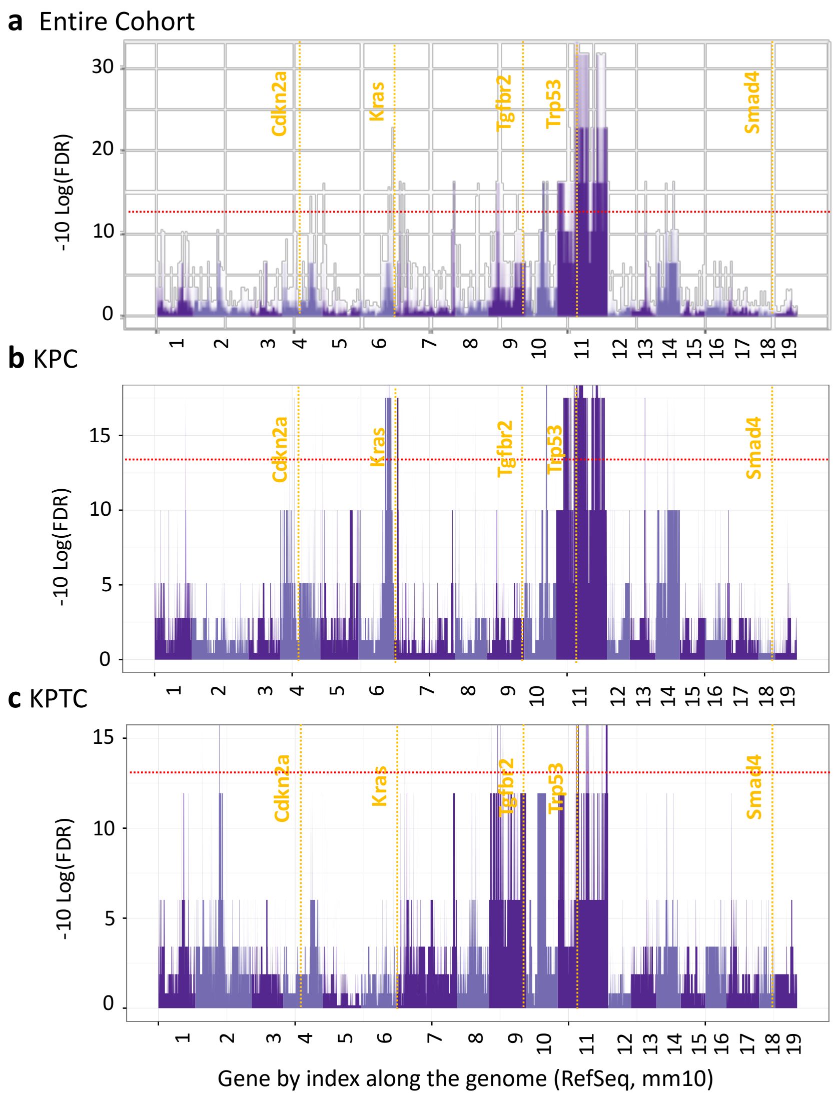

Supplementary Figure 3. Genome-wide significance for recurrent gene targets of allelic imbalance. Related to Figure 1c. In each mouse, a gene was marked as harboring allelic imbalance if such status was identified in at least one of the available tumor regions. Background rates of allelic imbalance were established across the entire cohort (a), the *KPC* mice (b), and the *KPTC* mice (c). Significance of the observed counts of allelic imbalance in a given gene was compared to the background rate and corrected for multiple hypothesis testing (BH). Significance levels are shown in Phred-scaled q-values. Red line marks FDR = 0.05.

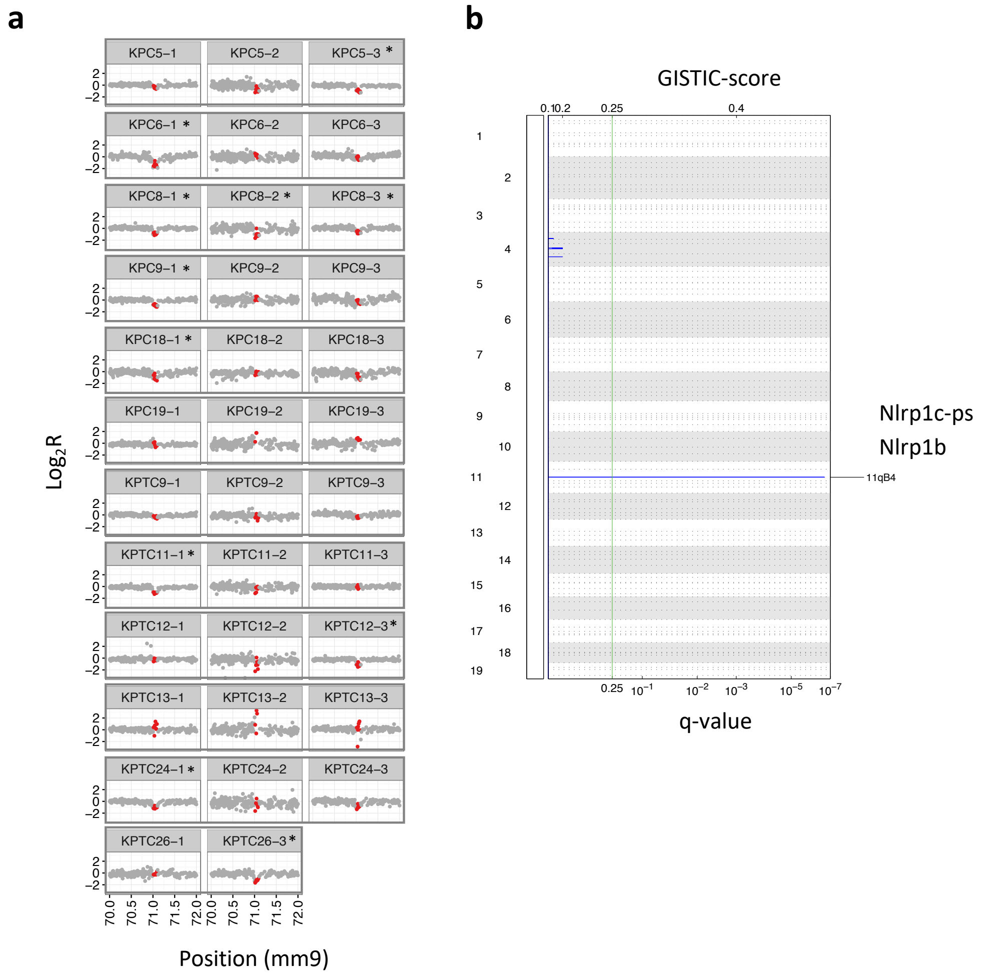

Supplementary Figure 4. Recurrent homozygous deletion in Nlrp1 locus on chr11. Related to Figure 1c. a. Log2 ratio of tumor to normal intensity for array probes in a 2Mb window centered at Nlrp1 locus. **b.** Genome-wide significance of focal copy number alterations. The only peak reaching genome-wide significance is the deletion of Nlrp1 locus on chr11qB4, overlapping Nlrp1b and Nlrp1c-ps. Asterisks indicate samples harboring the focal deletion.

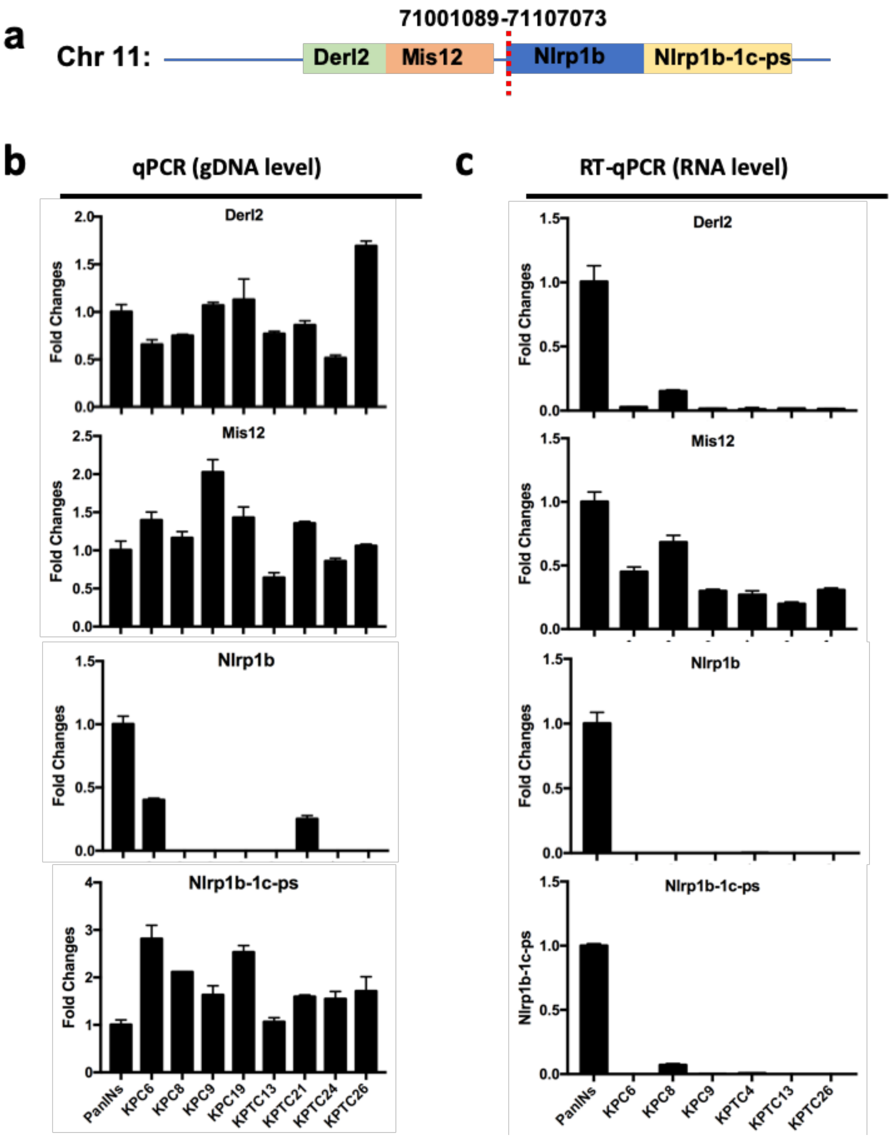

Supplementary Figure 5. Validation of a deletion mutation spanning chr11:71001089-71107073. a. A schematic diagram showing the relevant genes of the region. **b.** qPCR for amplifying a DNA fragment within the deleted locus (location indicated by dashed red line in panel A) as well as in two upstream and one downstream gene in five low passage cell lines established from *KPC* and *KPTC* tumors sequenced in this study. Genomic DNA extracted from mouse PanIN organoids were used as a control. **c.** qRT-PCR for expression of these same genes in these same tumor cell lines. Error bars represent the standard deviation of three independent measurements.

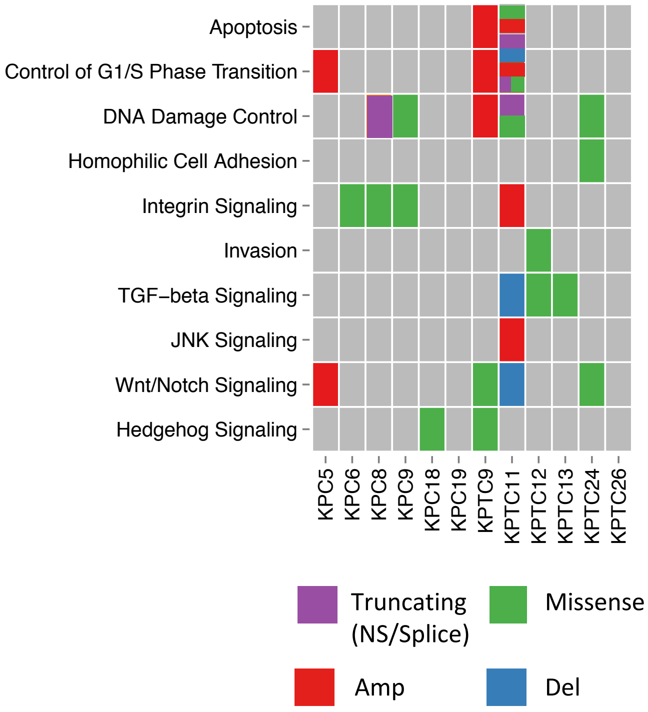

Supplementary Figure 6. Core pathways of human PDA and genomic alterations identified in six KPC and six KPTC mice in our study.
