# Supplementary material for: Characterization of genetic subclonal evolution in pancreatic cancer mouse models
Source: Nat Commun. 2019 Nov 28;10:5435. doi: 10.1038/s41467-019-13100-w (PMC6882784; doi:10.1038/s41467-019-13100-w)
Supplement: Supplementary file 2 — Description of Additional Supplementary Files [file 41467_2019_13100_MOESM2_ESM.pdf]

Description of Additional Supplementary Files

File Name: Supplementary Data 1
Description: Tumor Sample Characteristics

File Name: Supplementary Data 2
Description: Whole Exome Sequencing Summary

File Name: Supplementary Data 3
Description: Somatic Mutations

File Name: Supplementary Data 4
Description: Somatic Large Scale Copy Gains and Losses

File Name: Supplementary Data 5
Description: Exome-wide Allelic Imbalance Status and Enrichment Results

File Name: Supplementary Data 6
Description: Somatic Focal Amplifications and Deletions

File Name: Supplementary Data 7
Description: Significant Focal Copy Number Aberrations Identified By GISTIC

File Name: Supplementary Data 8
Description: Human Significantly Mutated Genes with Mutated Mouse Orthologs

File Name: Supplementary Data 9
Description: Pancreatic Cancer Core Pathways Altered by Mutation and Focal Copy Number Aberration in KPC and KPTC Mice

File Name: Supplementary Data 10
Description: Tumor Purity Estimated by Cre-Lox Sequence Analysis

File Name: Supplementary Data 11
Description: Somatic Mutation Cancer Cell Prevalence across Tumor Regions
