# Supplementary material for: Characterization of genetic subclonal evolution in pancreatic cancer mouse models
Source: Nat Commun. 2019 Nov 28;10:5435. doi: 10.1038/s41467-019-13100-w (PMC6882784; doi:10.1038/s41467-019-13100-w)
Supplement: Supplementary file 14 — Reporting Summary [file 41467_2019_13100_MOESM14_ESM.pdf]

nature research

Corresponding author(s): *Double-blind peer review submissions: write DBPR and your manuscript number here instead of author names.*

Last updated by author(s): *YYYY-MM-DD*

Reporting Summary

Nature Research wishes to improve the reproducibility of the work that we publish. This form provides structure for consistency and transparency in reporting. For further information on Nature Research policies, see Authors & Referees and the Editorial Policy Checklist.

Statistics

For all statistical analyses, confirm that the following items are present in the figure legend, table legend, main text, or Methods section.

n/a	Confirmed	
☐	☒	The exact sample size (*n*) for each experimental group/condition, given as a discrete number and unit of measurement
☒	☐	A statement on whether measurements were taken from distinct samples or whether the same sample was measured repeatedly
☐	☒	The statistical test(s) used AND whether they are one- or two-sided<br>*Only common tests should be described solely by name; describe more complex techniques in the Methods section.*
☒	☐	A description of all covariates tested
☐	☒	A description of any assumptions or corrections, such as tests of normality and adjustment for multiple comparisons
☒	☐	A full description of the statistical parameters including central tendency (e.g. means) or other basic estimates (e.g. regression coefficient) AND variation (e.g. standard deviation) or associated estimates of uncertainty (e.g. confidence intervals)
☐	☒	For null hypothesis testing, the test statistic (e.g. *F*, *t*, *r*) with confidence intervals, effect sizes, degrees of freedom and *P* value noted<br>*Give P values as exact values whenever suitable.*
☒	☐	For Bayesian analysis, information on the choice of priors and Markov chain Monte Carlo settings
☒	☐	For hierarchical and complex designs, identification of the appropriate level for tests and full reporting of outcomes
☒	☐	Estimates of effect sizes (e.g. Cohen's *d*, Pearson's *r*), indicating how they were calculated

Our web collection on statistics for biologists contains articles on many of the points above.

Software and code

Policy information about availability of computer code

Data collection	
Data analysis	CASAVA v1.8.2<br>GATK v3.1.1-g07a4bf8<br>Picard (v1.11)<br>MuTect (v1.1.7)<br>Ensemble Variant Effect Predictor tool<br>SIFT<br>VEST3<br>REVEL<br>Scalpel (v0.3.2)<br>TCGAbiolinks-2.2.10<br>SCHISM(1.1.2)

For manuscripts utilizing custom algorithms or software that are central to the research but not yet described in published literature, software must be made available to editors/reviewers. We strongly encourage code deposition in a community repository (e.g. GitHub). See the Nature Research guidelines for submitting code & software for further information.

Data

Policy information about availability of data

All manuscripts must include a data availability statement. This statement should provide the following information, where applicable:

- Accession codes, unique identifiers, or web links for publicly available datasets
- A list of figures that have associated raw data
- A description of any restrictions on data availability

Whole-exome and targeted sequence data of the KPC (n=6) and KPTC (n=6) tumor samples are available on the NCBI Short Read Archive database (Accession # PRJNA546566). Array CGH data of the same sample sets is available on the Gene Expression Omnibus database (Accession # GSE132235). All processed data relevant to this work are available in the Supplementary Files (Supplementary information, Description of Additional Supplementary Files and Supplementary Data 1-11). All other relevant data are available upon request.

Field-specific reporting

Please select the one below that is the best fit for your research. If you are not sure, read the appropriate sections before making your selection.

☒ Life sciences ☐ Behavioural & social sciences ☐ Ecological, evolutionary & environmental sciences

For a reference copy of the document with all sections, see nature.com/documents/nr-reporting-summary-flat.pdf

Life sciences study design

All studies must disclose on these points even when the disclosure is negative.

Sample size	Each tumor harvested from the pancreas of each mouse was divided into three distinct tumor regions, all three regions of each KPC (n=6) and KPTC (n=6) tumor and matched normal kidney tissue, In total of 48 samples were used for multi-region sequencing and copy number analyses.
Data exclusions	n/a
Replication	Instead of replication, we use multi-region sequencing strategy to capture both clonal and subclonal alterations.
Randomization	*Describe how samples/organisms/participants were allocated into experimental groups. If allocation was not random, describe how covariates were controlled OR if this is not relevant to your study, explain why.*
Blinding	*Describe whether the investigators were blinded to group allocation during data collection and/or analysis. If blinding was not possible, describe why OR explain why blinding was not relevant to your study.*

Reporting for specific materials, systems and methods

We require information from authors about some types of materials, experimental systems and methods used in many studies. Here, indicate whether each material, system or method listed is relevant to your study. If you are not sure if a list item applies to your research, read the appropriate section before selecting a response.

Materials & experimental systems

n/a	Involved in the study	
☒	☐	Antibodies
☐	☒	Eukaryotic cell lines
☒	☐	Palaeontology
☐	☒	Animals and other organisms
☒	☐	Human research participants
☒	☐	Clinical data

Methods

n/a	Involved in the study	
☒	☐	ChIP-seq
☒	☐	Flow cytometry
☒	☐	MRI-based neuroimaging

Eukaryotic cell lines

Policy information about cell lines

Cell line source(s)	KPC and KPTC cell lines were established at our lab
Authentication	Each cell lines were first authenticated by genotyping PCR, and further confirmed by examining the rearranged mutant allele(s) using recombination PCR strategy. Finally, the cells were also confirmed by confirming the growth of the grafted cells in the mice.
Mycoplasma contamination	Cell lines were confirmed mycoplasma free by using MycoAlertTM Mycoplasma Detection Kit (Lonza, LT07-318).

Commonly misidentified lines (See ICLAC register)	n/a

Animals and other organisms

Policy information about studies involving animals; ARRIVE guidelines recommended for reporting animal research

Laboratory animals	KPC (LSL-KRASG12D/+;LSL-Trp53R172H/+; Ptf1aCre/+) and KPTC (LSL-KRASG12D/+; LSL-Trp53R172H/+;Tgfbr2flox/-; Ptf1aCre/+) transgenic mouse models of PDA were generated in our lab as previously described {Hingorani, 2005;Zhong, 2017}. These mice are housed at the animal facility in MSKCC, and will be provided to other researchers upon request.
Wild animals	*Provide details on animals observed in or captured in the field; report species, sex and age where possible. Describe how animals were caught and transported and what happened to captive animals after the study (if killed, explain why and describe method; if released, say where and when) OR state that the study did not involve wild animals.*
Field-collected samples	*For laboratory work with field-collected samples, describe all relevant parameters such as housing, maintenance, temperature, photoperiod and end-of-experiment protocol OR state that the study did not involve samples collected from the field.*
Ethics oversight	*Identify the organization(s) that approved or provided guidance on the study protocol, OR state that no ethical approval or guidance was required and explain why not.*

Note that full information on the approval of the study protocol must also be provided in the manuscript.
